# Synthesis, Antiviral, and Antimicrobial Evaluation of Benzyl Protected Diversified *C*-nucleosides

**DOI:** 10.3389/fchem.2018.00294

**Published:** 2018-07-19

**Authors:** Ahmed Bari, Syed S. Ali, Amer M. Alanazi, Muhammad A. Mashwani, Abdulrahman M. Al-Obaid

**Affiliations:** ^1^Department of Pharmaceutical Chemistry, College of Pharmacy, King Saud University, Riyadh, Saudi Arabia; ^2^Research Centre, College of Pharmacy, King Saud University, Riyadh, Saudi Arabia; ^3^Department of Pathology and Microbiology, King Khalid University Hospital, King Saud University, Riyadh, Saudi Arabia

**Keywords:** formyl glycal, formyl rhamnal, nucleosides, villsmeir-haack, ring transformation

## Abstract

Formyl glycals are the versatile synthetic intermediates and can serves as precursor for the synthesis of various C and N-nucleosides. Due to the presence of electron donating and electron withdrawing character on formyl sugars which makes the molecule more susceptible to nucleophilic attack. Utilizing same strategy, we propose the synthesis of diversified *C*-nucleosides (**3**-**14**) by reaction with *N*,*N* dinucleophiles. These nucleoside analogs were than tested against viral, bacterial and fungal strains.

## Introduction

Carbohydrates being the important part of the wide variety of biological functions and consequently show enormous potential as therapeutic agents. After the discovery of several potent nucleosides like acyclovir, biopterin A and bengazol, substantial efforts have been made to the synthesis of less susceptible, more active nucleoside analogs. It was also known that carbohydrates and their derivatives play an important biological role in all living matter and functions. Several polycyclic and fused sugars starting from hexose and pentose glycals, have been reported which were used as synthetic intermediates to synthesize a variety of nucleoside analogs (Bari, 2014). All these endocyclic molecules serve as excellent precursors for ring transformation reactions with various *N*-nucleophiles leading to different types of heterocyclic and carbocyclic annelated nucleosides (Bari et al., 2005a).

Due to the rapid development of drug resistance, new antibacterial and antiviral agents should be designed and synthesized with the chemical properties clearly different from those of existing agents. Nitrogen-containing heterocyclic molecules are well known chemical entities, which are part of many naturally occurring products and pharmaceuticals vital for the life. Carbohydrate moiety attached to the heterocyclic system can be expected to play a role as drug carrier, hence improves the pharmacikinetics of drugs.

In our previous studies formyl glycals and push-pull butadienes when reacted with *N*,*N*-binucleophiles, afforded pyrazole, pyridine, and pyrimidine acyclo-*C*-nucleosides (Bari et al., 2004). Because of the biological potential of this class of compounds and in accordance with our efforts on exploring the new methodologies, we now describe the transformations of benzyl protected 2-formylglucal and rhamnal in a process to obtain diversified *C*-nucleoside analogs which were never reported before. Moreover, antibacterial, and antiviral potential of these molecules has also been exploited. Pyrazoles constitute an important class of compounds in organic synthesis due to two nitrogen groups being part of the molecule. In addition, pyrazolo-pyrimidines are considered as one of the most useful synthetic intermediate to synthesize variety of molecules and possible drug candidates (Hocková et al., 1999; Quiroga et al., 2009). Since many years, hydrazide derivatives have been the focus of interest for many synthetic chemists and biologists because of the synthetic importance and the biological activity associated with them. The pharmacological profile includes their antimicrobial, antiviral, anticancer and anti-inflammatory activities (Thiele et al., 2005). The bioactivity of the hydrazide analogs is apparently not limited to the core moiety but also depends on the molecules attached to the terminal nitrogen. The current paper describes the synthesis of diversified acyclo-*C*-nucleosides and their antiviral and anti microbial evaluation.

Push-pull activated formyl glycal and rhamnal, synthesized by the Vilsmeier-Haack reaction are the versatile synthetic intermediates due to the presence of electron donating and withdrawing groups making the molecule susceptible to nucleophilic attack (Ramesh and Balasubramanian, 1991). Ring transformation involving formyl group resulted in a formation of nucleoside analogs with antiviral properties.

**Scheme 1:**
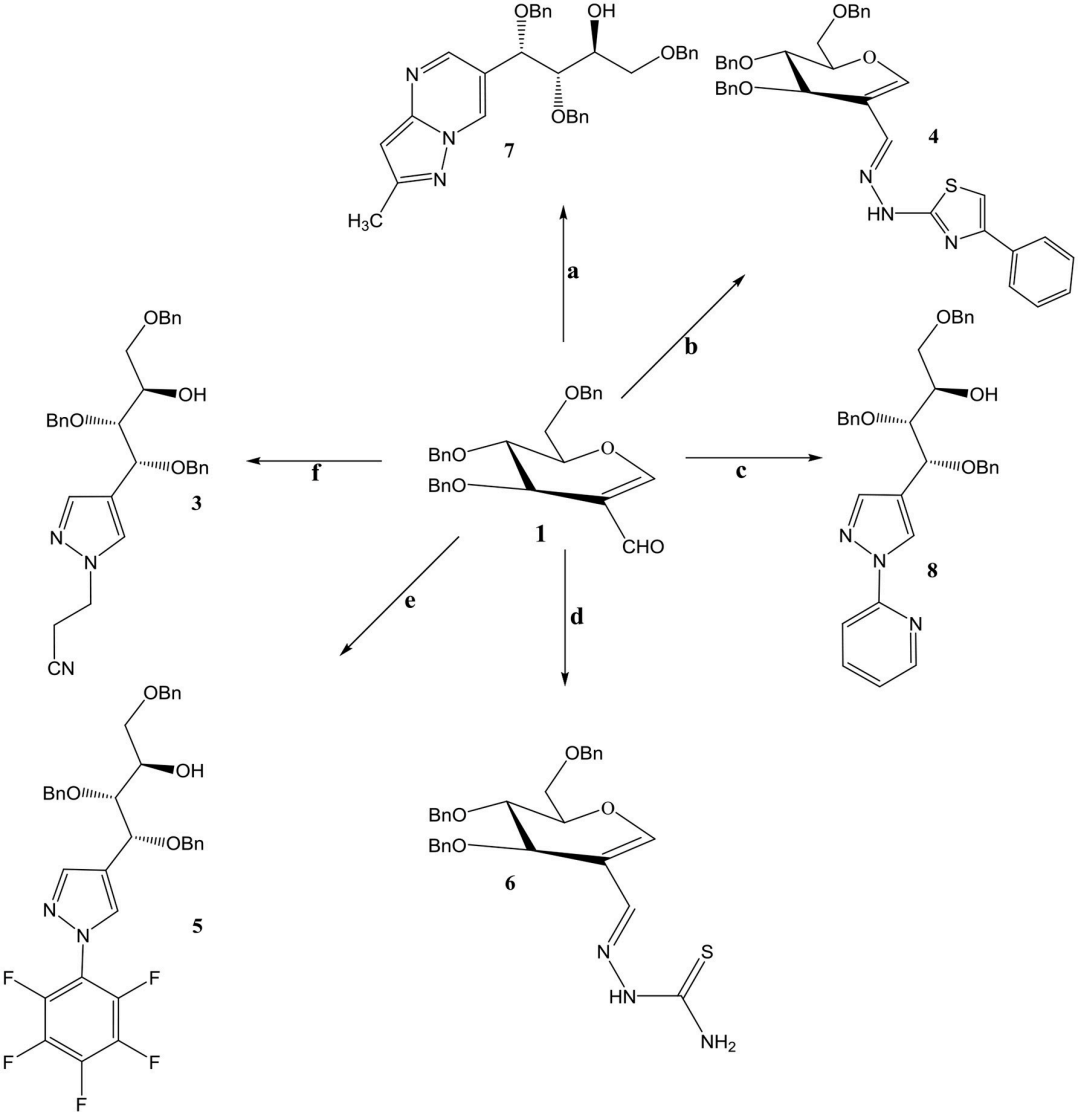
Reagents and condition: Ethanol, reflux, OBn =-CH2Ph. **a**: 3-methyl-1H pyrazole-5-amine. **b**: 2-hydrazino-4-phenylthiazole **c**: 2-hydrazinopyridine **d**: Thiosemicarbazide. **e**: Pentaflurophenyl hydrazine **f**: Cyanoethyl hydrazine.

**Scheme 2:**
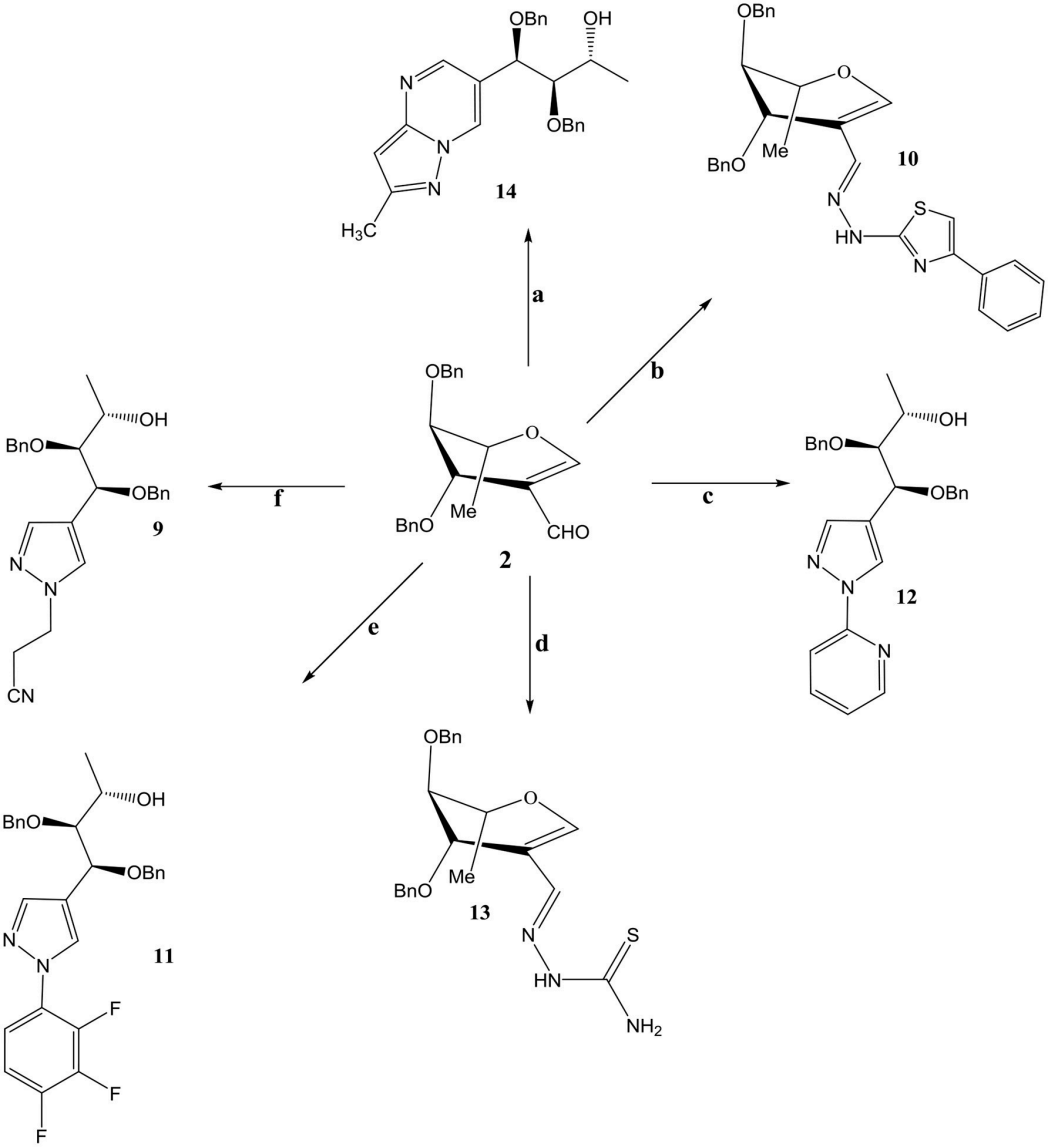
Reagents and condition: Ethanol, reflux, OBn =-CH2Ph. **a**: 3-methyl-1H pyrazole-5-amine. **b**: 2-hydrazino-4-phenylthiazole **c**: 2-hydrazinopyridine **d**: Thiosemicarbazide. **e**: Pentaflurophenyl hydrazine **f**: Cyanoethyl hydrazine.

**Scheme 3:**
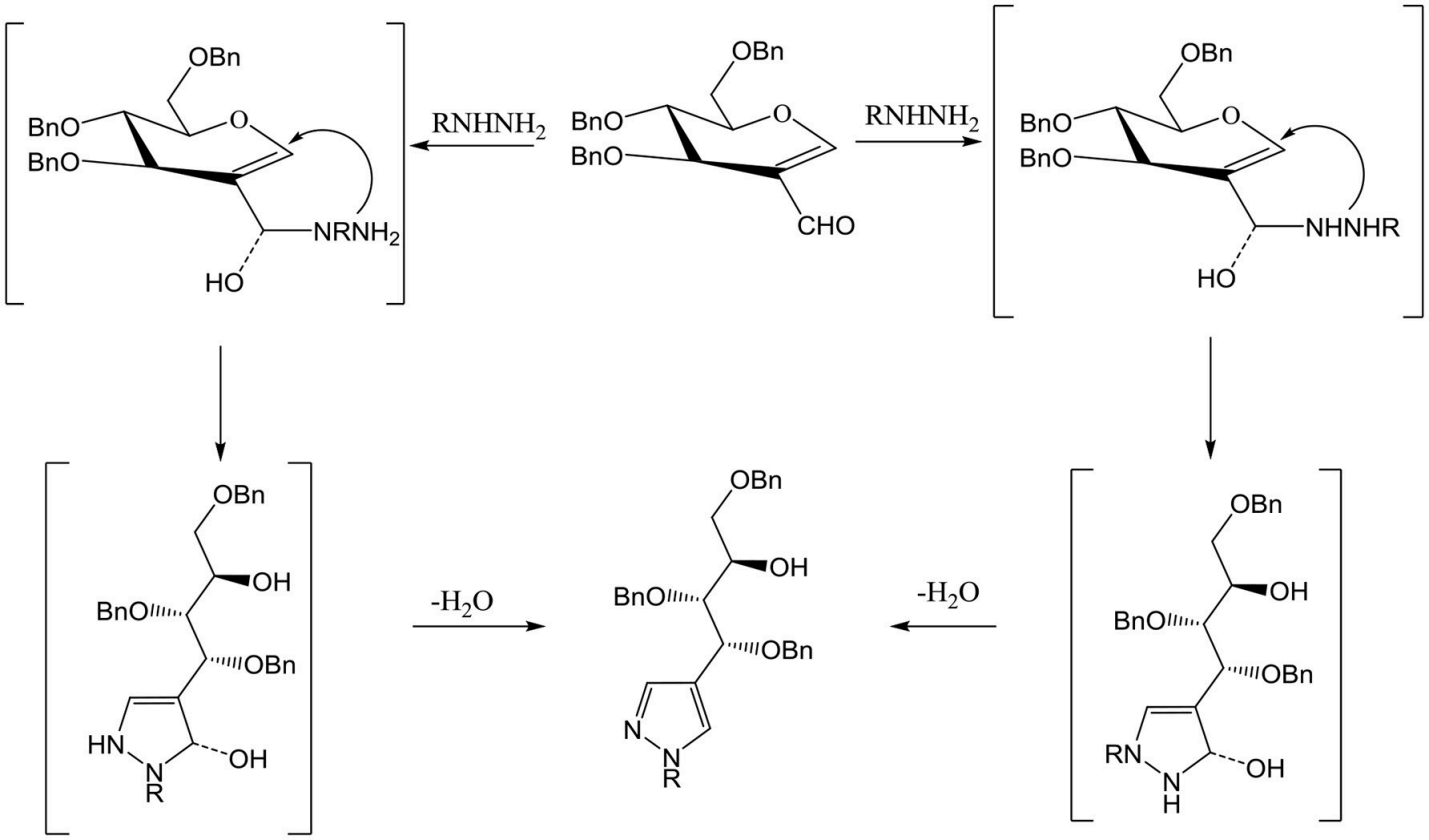
Proposed mechanism for the synthesis of pyrazole-ayclo-*C*-nucleosides.

Compound **3** and **9** were synthesized according to an inventive method in which formyl bearing sugars **1** and **2** were reacted with cyanoethylhydrazine in refluxing ethanol afforded pyrazole-acyclo-*C*-nucleosides (Scheme 1). Presumably, the reaction proceed by the nucleophilic attack of hydrazine which acts as dinucleophiles at C-1 which resulted in the ring opening of glycals followed by the cyclization involving formyl group (Scheme 3). The products were confirmed by ^1^H and ^13^C NMR spectroscopy in which the spectra of these compounds showed the absence of signals for formyl group instead the typical long range coupling of pyrazole protons were observed confirming the successful ring transformation (Montero et al., 2004; Bari et al., 2005b). The products were purified by column chromatography and were obtained in quantitative yield with no side products formed. Moreover, in the attempt of finding new acyclo-*C*-nucleoside with the attached phenylthiazole moiety **1** and **2** were reacted with 2-hydrazine-4-phenylthiazole in refluxing ethanol but even after longer reaction time the ring transformation was not observed instead hydrazone **4** and **10** were formed (Scheme 2). This could be due to the steric hindrance of the attached phenylthiazole group making the attached nitrogen less likely to attack at C-1. The products were confirmed by NMR spectroscopy where the absence of formyl group and the presence of C-1 and C-1' more downfield in the ^1^H and ^13^C NMR spectroscopy which is a typical push-pull behavior (Rudloff et al., 2002). Moreover, the absence of pyrazole protons further confirmed that no ring transformation occurs. With gated decoupling it could be proved that only *E*-isomers were obtained.

The stability of C-F bond and its small size makes it possible to replace any C-H bond in organic molecule. It is also a well-known fact that nucleosides having fluoro group penetrates easily in to the enzyme pockets without causing any steric hinderance (Koshida et al., 1989). Moreover, due to the strong electron-withdrawing effect on C-F bond induces electrostatic intramolecular.

interactions therefore changes the conformation of molecules, making it more interesting than the conventional ones. In continuation of our search for more potent nucleosides compounds **1** and **2** were reacted in refluxing ethanol with pentafluoro phenylhydrazine to produce **5** and **11** in excellent yields. Presence of two pyrazole protons giving coupling constant of 2.1 Hz which is a characteristic of *W* type coupling confirms the elucidated structures. Mass and elemental analysis are also in accordance with the proposed data.

Reaction of 2-formyl glycal **1** and 2-formylrhamnal **2** when reacted with thiosemicarbazide afforded semicarbazones **6** and **13** respectively, in good to moderate yields. NMR spectroscopy confirmed the formation of *E* isomers which is a most favored confirmation and reported in similar type of reactions (Gumina and Chu, 2003; Tenchiu et al., 2009). An excellent example of the reactivity of *N*-*N* binucleophiles is attributed in the reaction of **1** and **2** with 3-methyl-1*H*-pyrazole-5-amine yielding **7** and **14** respectively. The structures were confirmed by NMR and mass spectroscopy which are in accordance with the proposed molecules.

## Materials and methods

### Biology

#### Antiviral data collection

The antiviral assays were based on inhibition of virus-induced cytopathogenicity or plague formation in selective virus infected cell lines. Three different laboratory strains of viruses were used in the present study which are the following, herpes simplex virus type 1 (HSV-1-KOS), varicella-Zoster virus (VZV) and human cytomegalo virus (HCMV) to evaluate antiviral potential of the synthesized molecules. The reason for selecting HSV strain KOS is because it has been widely used in many research studies to study HSV-1 replication, gene expression, and pathogenesis (Clayette et al., 1991 and Kodama et al., 2001; Montagu et al., 2011). Moreover, the HSV-1 KOS strain has been known to be less virulent, less pathogenic, and has better ability to attach to the host cells compared to other HSV strains and can also produce bigger plaques and can better associate to host cells via attachment, which means virus particles that endure attachment to or within the host cell once replicated. Varicella zoster virus (VZV) obtained from a zoster vesicle is highly cell-associated, and was propagated by co-cultivation of infected cells with uninfected cells at ratios depending on use (Furman et al., 1995; Wilson et al., 1996).

The antiviral assays were based on plaque formation in all three type of viral strains used. For the simultaneous assay, HSV-1, HCMV, and VZV cells were grown in 96-well plates for 24 h at 37°C with 5% CO_2_ in air. Plates were inoculated with 100 CCID_50_ of viruses where 1 CCID_50_ being the virus dose to infect 50% of the cell cultures. The residual virus was removed after adsorption period which is of 2 h. The cell cultures were than incubated with different concentrations of test compounds (100 μM, 30 μM). Cytopathicity of control virus-infected cell cultures which were not treated with test compounds were recorded immediately when it reached completion. Antiviral activity was recorded as EC_50_ values which was defined as the concentration that reduced the absorbance of infected cells to 50% when compared to infected cells and control cells or concentration required reducing virus-induced cytopathogenicity by 50%. The minimal cytotoxic concentration (MCC) of the compounds was defined as the compound concentration that caused a microscopically visible alteration of cell morphology. The measurement of cytotoxicity of test compounds was recorded on the basis of their cell growth inhibition potency. 96-well microtitre plates were used for the sedation of HEL cells at a rate of 5 × 10^2^ for 24 h. The medium containing different concentrations of test compounds were then added followed by the incubation at 37° for 3 days. Coulter counter was used to determine the cell numbers (Table 1).

**Table 1:** Antiviral Activity of *C*-nucleosides against DNA viruses.

**Compounds**	**^a^HSV-1 (KOS)**	**^b^VZV (TK^+^)**	**^c^HCMV (AD-169)**	**^d^MCC**	**^e^EC-_50_^(μ*M*)^**
2	61	>110	50	110	170
3	11	51	>100	77	49
4	49	71	90	>100	180
5	>100	>200	>200	>200	70
6	99	60	>200	140	100
7	72	89	-	88	>100
8	19	33	41	21	41
9	33	61	70	56	60
10	54	59	-	220	>100
11	73	101	140	98	110
12	>100	70	-	>50	67
13	49	72	77	71	-
14	-	105	90	>100	-
15	21	>300	-	49	55
Ganciclovir	0.02	0.7	-	>100	0.5
Acyclovir	0.5	1.1	-	>50	2

a *HSV is Herpes Simplex virus type 1*.

b *VZV is varicella-Zoster virus*.

c *HCMV is human cytomegalo virus*.

d *Minimum cytotoxic concentration (MCC) that causes a microscopically detectable change in cell morphology*.

e *Effective concentration (EC-50 in μM) required to reduce virus-induced cytopathicity or viral plague by 50%*.

#### Antibacterial studies

##### Sample preparation

Samples were stored frozen at −20°C, prepared in DMSO and water to a final testing concentration of 32 μg/mL or 20 μM (unless otherwise indicated in the Data Sheet), in 384-well, non-binding surface plate (NBS) for each bacterial/fungal strain, and in duplicate (*n* = 2), and keeping the final DMSO concentration to a maximum of 1% DMSO. All the sample preparation was done using liquid handling robots. For detailed information, see the Supplementary Material.

#### Antibacterial assay

##### Procedure

All bacterial strains obtained from ATCC (SA: ATCC 4330, EC: ATCC 25922, KP: ATCC 700603, AB: ATCC 19606, PA: ATCC 27853) were cultured in Cation-adjusted Mueller Hinton broth (CAMHB) at 37°C overnight. A sample of each culture was then diluted 40-fold in fresh broth and incubated at 37°C for 1.5–3 h. The resultant mid-log phase cultures were diluted (CFU/mL measured by OD_600_), then added to each well of the compound containing plates, giving a cell density of 5 × 105 CFU/mL and a total volume of 50 μL. All the plates were covered and incubated at 37°C for 18 h without shaking.

##### Analysis

Inhibition of bacterial growth was determined by measuring absorbance at 600 nm (OD_600_), using a Tecan M1000 Pro monochromator plate reader. The percentage of growth inhibition was calculated for each well, using the negative control (media only) and positive control (bacteria without inhibitors) on the same plate as references. Samples with inhibition value 80% and *Z*-Score 2.5 for either replicate (*n* = 2 on different plates) were classed as actives. Samples with inhibition values between 50 and 80% and *Z*-Score above 2.5 for either replicate (*n* = 2 on different plates) were classed as partially actives. Samples with inhibition values between 50 and 80% and *Z*-Score above 2.5 for either replicate (*n* = 2 on different plates) were classed as partial actives (Table 2).

**Table 2:** Antimicrobial evaluation data of synthesized compounds.

**Compound**	**Sa**	**Ec**	**Kp**	**Pa**	**Ab**	**Ca**	**Cn**
1	A	I	I	I	A	I	A
2	I	I	I	I	A	I	I
3	I	I	I	I	I	I	I
4	I	I	I	I	I	I	I
5	I	I	I	I	P	A	A
6	I	I	I	I	I	A	A
7	I	I	I	I	I	I	I
8	I	I	I	I	A	I	I
9	I	I	I	I	P	I	I
10	I	I	I	I	P	A	A
11	I	I	I	I	I	A	A
12	I	I	I	I	I	I	I
13	I	I	I	I	I	I	I
14	I	I	I	I	I	I	I
Tri-O-benzyl-D-glucal	I	I	I	I	I	I	I

*A, active; I, inactive; P, partially active*.

*Sa is Staphylococcus aureus, Ec is Escherichia coli, Kp is Klebsiella Pneumoniae, Pa is Pseudomonas aeruginosa*.

*Ab is Acinetobacter baumannii, Ca is Candida Albicans, Cn is Cryptococcus neoformans var. grubii*.

#### Antifungal assay

##### Procedure

Fungi strains obtained from ATCC (CA: ATCC 90028, CN: ATCC 208821) were cultured for 3 days on Yeast Extract-Peptone Dextrose (YPD) agar at 30°C. A yeast suspension of 1 × 10^6^ to 5 × 10^6^ CFU/mL (as determined by OD_530_) was prepared from five colonies. The suspension was subsequently diluted and added to each well of the compound-containing plates giving a final cell density of fungi suspension of 2.5 × 10^3^ CFU/mL and a total volume of 50 μL. All plates were covered and incubated at 35°C for 24 h without shaking.

##### Analysis

Growth inhibition of *C. albicans* was determined measuring absorbance at 530 nm (OD_530_), while the growth inhibition of *C. neoformans* was determined measuring the difference in absorbance between 600 and 570 nm (OD_600−570_), after the addition of resazurin (0.001% final concentration) and incubation at 35°C for additional 2 h. The absorbance was measured using a Biotek Synergy HTX plate reader. The percentage of growth inhibition was calculated for each well, using the negative control (media only) and positive control (bacteria without inhibitors) on the same plate.

Samples with inhibition value above 80% and *Z*-Score above 2.5 for either replicate (*n* = 2 on different plates) were classed as actives. Samples with inhibition values between 50 and 80% and *Z*-Score above 2.5 for either replicate (*n* = 2 on different plates) were classed as partial actives.

##### Quality control

Antibiotics, Colistin and Vancomycin were used as positive bacterial inhibitor controls for Gram-negative and Gram-positive bacteria, respectively. Fluconazole was used as a positive fungal inhibitor control for *C. albicans and C. neoformans*. The antibiotics were provided in 4 concentrations, with 2 above and 2 below its MIC (minimum inhibitory concentration) value, and plated into the first 8 wells of column 23 of the 384-well NBS plates. All screening are done as two replica (*n* = 2), with both replicas on two different plates, but from single plating and done in a single screening (microbial incubation). Each individual value is reported in the Table 1. In addition, two values are used as quality control for individual plates. The plate passes the quality controls if Z-Factor >0.4 and standards are active and inactive at highest and lowest concentrations, respectively.

## Discussion

Fifteen nucleoside analogs including two starting materials have been tested against three viral, five bacterial and two fungal strains. Our experimental results with *C*-nucleosides demonstrated the absence of significant antiviral effect, although some of them showed inhibition to some extent but the overall results are not significant. It is obvious that the synthesized molecules are not specific reverse transcriptase inhibitors as compared to the reference drugs. This might be due to the benzyl protecting groups which may cause lower solubility hence less available to the virus. Acyclo-*C*-nucleosides are either not cytotoxic or are slightly so similarly to the compounds with exocyclic push-pull butadienes. Only compound **8** and **9** induced cellular toxicity for the viral strains used but the toxicity induced by them is much lower than the two controls used for comparison. Compound 4 also showed slight toxicity against HSV-type 1 but are almost inactive against other strains used. Moreover, the less cytotoxicity of acyclic nucleosides is attributed to the small carbon chain which may not be phosphorylated by cellular kinases when enter into the cells.

Synthesized compounds tested against 5 bacterial and two fungal strains were mostly active. Samples with inhibition value above 80% were classed as actives. Samples with inhibition values between 50 and 80% were classed as partial actives while the samples with inhibition values between 50 and 80% were classed as partial actives. Compounds 1 and 2 showed activity against acinetobacter baumannii while 5, 6, 8, and 9 showed some cytotoxicity against candida albicans and Cryptococcus neoformans.

In summary, the present results demonstrated the activity of nucleosides as antimicrobial agents. Moreover, the synthesized nucleosides showed no activity against the selected viral strains which further support the hypothesis that protecting groups on nucleosides plays an important role in biological processes.

### Chemistry

All solvents and reagents were purchased from Aldrich Chemical Co and were used as obtained. IR spectra were recorded with a Bruker alpha FT-IR spectrometer. ^1^H- and ^13^C-NMR spectrum was recorded on a Bruker instrument at 500, 700, and 125 MHz resp, at 293 K in DMSO-*d*_6_ and or CDCl_3_ using TMS as internal standard. The mass spectrometric analyses were carried out on a Micromass LCT mass spectrometer. The elemental analyses for C, H, and N were done on Perkin-Elmer CHN-2440 analyzer (C, H, N) and were in full agreement with the proposed structures. Thin-layer chromatography (TLC) was performed on a fluorescent aluminum backed silica gel HF^254^ plates (Merck) and was viewed under UV 254 and 265 lights and charring with EtOH/H_2_SO_4_. Merck silica gel 60 (230–400 mesh) was used for column chromatography separations with toluene and ethylacetate as eluents.

### General procedure for the synthesis of compounds 3-14

A mixture containing the formyl glycal or rhamnal (1 mmol) in anhydrous ethanol was added appropriate nucleophile (1.1 mmol) at 0°C for 10 min with stirring. The mixture was than heated under reflux for 1 hr. After completion of the reaction, as indicated by TLC, the reaction mixture was evaporated and the residue purified by column chromatography. In most of the cases the required products was eluted in the range of toluene/ethylacetate 1:1 as syrups.

### 3-(4-((1S,2S,3S)-1,2,4-tris(benzyloxy)-3-hydroxybutyl)-1H-pyrazol-1-YL)propanenitrile (3)

Yield: 51 %, yellowish syrup, IR (NaCl): *v* = 1722 (C = O), 2221 (C = N): ^1^HNMR (500.174 MHz, CDCl_3_): δ = 1.97 (br s, OH), 3.2 (m, 2H, CH_2_), 3.47 (dd, 1H, *j* = 7.1 Hz CH), 3.60 (m, 1H, CH), 3.69 (m, 1H, CH), 3.81 (m, 2H, 2 × CH), 4.35 (m, 2H, CH), 4.37 (m, 6H, 3 × CH_2_), 7.27-7.37 (m, 15H, Ph), 7.45 (s, 1H, CH), 7.60 (s, 1H, CH). ^13^C NMR (125.76 MHz, CDCl_3_): δ = 19.3, 31.4, 36.5, 47.5, 70.2, 70.9, 73.0, 73.4, 73.7, 74.3, 81.0, 82.4, 114.1, 120.1, 127.8-128.4 (13 × C), 135.5, 136.1, 137.9, 162.5. MS (CI isobutane): *m/z* (%) = 511 (11) (M+). – C_31_H_33_N_3_O_4_ (480): calcd. C 72.78, H 6.50, N 8.21 found C 72.69, H 6.63, N 8.19.

### 2-((E)-2-(((2R,3S)-3,4-bis(benzyloxy)-2-(benzyloxymethyl)-3,4-dihydro-2H-pyran-5-yl)methylene)hydrazinyl)-4-phenylthiazole) (4)

Yield: 53 %, yellowish syrup, IR (NaCl): *v* = 1718 (C = O), 3030 (NH): ^1^HNMR (500.174 MHz, CDCl_3_): δ = 3.70 (dd, 2H, *j* = 10.1 Hz, *j* = 4.9 Hz, CH_2_), 3.81 (t, 1H, CH), 4.44 (d, 2H, *j* = 11.2 Hz, CH_2_), 4.53 (d, 2H, *j* = 11.7 Hz, CH_2_), 4.55 (m, 2H, 2 × CH), 4.64 (m, 2H, 2 × CH), 6.11 (s, 1H, CH), 6.93 (s, 1H, CH), 7.30-7.43 (m, 21H, Ph), 11.10 (s, 1H, NH). ^13^C NMR (125.76 MHz, CDCl_3_): δ = 67.7, 68.4, 70.9, 71.5, 71.8, 102.6, 110.9, 126.2 (2 × C), 126.3-128.8 (20 × C), 134.9, 137.5, 137.8, 138.5, 142.5, 149.4, 151.1, 169.9. MS (CI isobutane): *m/z* (%) = 617 (21) (M+). – C_37_H_35_N_3_O_4_ S (480): calcd. C 71.94, H 5.71, N 6.80, S 5.19 found C 71.84, H 5.83, N 6.88, S 5.10.

### (2S,3S,4S)-1,3,4-tris(benzyloxy)-4-(1-(perfluorophenyl)-1H-pyrazol-4-yl)butan-2-ol (5)

Yield: 59 %, yellowish syrup, IR (NaCl): *v* = 1722 (C = O), 2221 (C = N): ^1^HNMR (500.174 MHz, CDCl_3_): δ = 1.93 (br s, OH), 3.29 (m, 2H, CH_2_), 3.51 (dd, 1H, *j* = 6.8 Hz, CH), 3.66 (m, 1H, CH), 3.88 (m, 2H, 2 × CH), 4.47 (m, 4H, 2 × CH_2_), 4.73 (d, 1H, CH), 7.21-7.30 (m, 15H, Ph), 7.41 (s, 1H), 7.59 (s, 1H). ^13^C NMR (125.76 MHz, CDCl_3_): δ = 70.2, 70.9, 73.0, 73.4, 73.7, 74.3, 81.0, 82.4, 120.1, 127.8-128.4 (16 × C), 135.5, 135.9, 136.1, 136.6, 137.3, 137.9, 138.5, 138.9, 162.5. MS (CI isobutane): *m/z* (%) = 624 (33) (M+). –C_34_H_29_F_5_N_2_O_4_ (624): calcd. C 65.38, H 4.68, N 4.49 found C 65.49, H 4.63, N 4.28.

### (E)-2-(((2R,3S)-3,4-bis(benzyloxy)-2-(benzyloxymethyl)-3,4-dihydro-2H-pyran-5-yl)methylene)hydrazinecarbothioamide (6)

Yield: 42 %, yellowish syrup, IR (NaCl): *v* = 1610 (C = N), 3200 (NH): ^1^HNMR (500.174 MHz, CDCl_3_): δ = 3.69 (m, 2H, CH_2_), 3.80 (dd, 2H, 2 × CH), 4.51 (d, 2H, *j* = 10.9 Hz, CH_2_), 4.55 (d, 2H, *j* = 11.2 Hz, CH_2_), 4.58 (m, 1H, CH), 4.62 (d, 1H, CH), 6.10 (s, 1H, CH), 6.78 (s, 1H, CH), 7.21-7.37 (m, 16H, Ph, CH), 9.12 (s, 1H, NH), 11.10 (s, 2H, NH_2_). ^13^C NMR (125.76 MHz, CDCl_3_): δ = 67.72 67.9, 69.7, 70.5, 71.6, 101.9, 111.2, 125.8 (3 × C), 126.1-128.8 (12 × C), 134.5, 136.9, 137.8, 140.1, 143.7, 149.5, 189.2. MS (CI isobutane): *m/z* (%) = 518 (11) (M+). –C_29_H_31_N_3_O_4_S (517.20): calcd. C 67.29, H 6.04, N 8.12, S 6.19 found C 67.34, H 5.93, N 8.18, S 6.10.

### (2R,3R,4R)-1,3,4-tris(benzyloxy)-4-(2-methylpyrazolo[1,5-a]pyrimidin-6-yl)butan-2-ol (7)

Yield: 49 %, yellowish syrup, IR (NaCl): *v* = 1630 (C = N), 3377 (OH): ^1^HNMR (500.174 MHz, CDCl_3_): δ = 2.31 br s (OH), 2.56 (s, 3H, Me), 3.60 (dd, 1H, *j* = 3.0 Hz, CH); 3.69 (d, 2H, *j* = 4.0 Hz, CH_2_); 4.08 (m, 1H, CH); 4.2 (d, 1H, *j* = 11.5 Hz, CH); 4.35 (d, 2H, *j* = 12.0 Hz, CH_2_); 4.55 (s, 2H, 2 × CH); 4.61 (d, 1H, *j* = 11.5 Hz, CH); 4.76 (d, 1H, *j* = 11.5 Hz, CH); 7.01 (m, 2H, Ph); 7.11 (m, 3H, Ph); 7.32 (m, 10H, Ph); 8.11 (s, 1H, CH), 8.40 (s, 1H, CH); 8.50 (s, 1H, CH); ^13^C NMR (125.76 MHz, CDCl_3_): δ = 13.5, 68.8, 69.6, 70.6, 72.5, 73.4, 75.0, 79.9, 94.9, 127.4–128.9 (15 × C); 132.2, 135.8, 135.9, 136.5, 147.9, 148.3, 154.3, 155.1. MS (CI isobutane): *m/z* (%) = 522 (39) (M+). –C_32_H_33_N_3_O_4_ (523.25): calcd. C 73.40, H 6.35, N 8.02, found C 73.33, H 6.21, N 8.15.

### (2R,3R,4R)-1,3,4-tris(benzyloxy)-4-(1-(pyridin-2-yl)-1H-pyrazol-4-yl)butan-2-ol (8)

Yield: 59 %, yellowish syrup, IR (NaCl): *v* = 1622 (C = N), 3364 (OH): ^1^HNMR (500.174 MHz, CDCl_3_): δ = d 2.71 (br, 1H, OH); 3.47 (m, 2H, CH_2_); 3.55 (m, 1H, CH); 3.83 (m, 1H, CH); 4.10 (d, 1H, *j* = 11.2 Hz, CH); 4.29 (d, 1H, *j* = 11.2 Hz, CH); 4.32 (m 2H, CH_2_); 4.39 (m, 2H, CH_2_); 4.43 (m, 2H, CH_2_); 4.55 (d, 1H, CH); 7.11–7.17 (m, 2H, Ph); 7.21–7.30 (m, 13H, Ph); 7.33 (d, 1H, CH); 7.47 (d, 1H, *j* = 1.2 Hz, CH); 7.91 (dd, 1H, CH); 8.27 (d, 1H, *j* = 2.4 Hz, CH). ^13^C NMR (125.76 MHz, CDCl_3_): δ = 71.1, 71.3, 71.9, 72.8, 73.1, 74.9, 79.7, 120.3, 121.6, 127.1–128.7 (16 × C), 129.3, 129.8, 136.9, 137.5, 137.9, 139.0, 148.3, 149.8. MS (CI isobutane): *m/z* (%) = 535 (39) (M+). –C_33_H_33_N_3_O_4_ (535.25): calcd. C 74.00, H 6.21, N 7.84, found C 73.93, H 6.29, N 8.01.

### 3-(4-((1S,2S,3S)-1,2-bis(benzyloxy)-3-hydroxybutyl)-1H-pyrazol-1-yl)propanenitrile (9)

Yield: 46 %, yellowish syrup, IR (NaCl): *v* = 1570 (C = C), 2223 (C = N): ^1^HNMR (500.174 MHz, CDCl_3_): δ = 1.10 (d, 3H, *j* = 6.5 Hz, Me), 3.3 (t, 2H, CH_2_), 3.55 (dd, 1H, CH), 3.71 (m, 1H, CH), 4.20 (d, 1H, CH), 4.32 (m, 2H, CH_2_), 4.36 (d, 1H, *j* = 5.5 Hz, CH), 4.40 (d, 1H, *j* = 12.1 Hz, CH), 4.92 (t, 2H, *j* = 10.2 Hz, CH_2_), 8.05 (d, 1H, *j* = 2.2 Hz, CH). ^13^C NMR (125.76 MHz, CDCl_3_): δ = 14.70, 18.64, 50.10, 67.20, 71.77, 72.69, 83.76, 90.84, 116.30, 126.1-127.5 (10 × C), 120.10, 124.71, 129.24, 138.2, 139.7. MS (CI isobutane): *m/z* (%) = 405.21 (21) (M+). – C_24_H_27_N_3_O_3_ (405): calcd. C 71.09, H 6.71, N 10.36 found C 69.99, H 6.66, N 10.38.

### 2-((E)-2-(((2S,3S,4S)-3,4-bis(benzyloxy)-2-methyl-3,4-dihydro-2H-pyran-5-yl)-methylene)hydrazinyl)-4-phenylthiazole (10)

Yield: 49 %, yellowish syrup, IR (NaCl): *v* = 3010 (NH), 3230 (OH) ^1^HNMR (500.174 MHz, CDCl_3_): δ = 1.39 (d, 3H, *j* = 7.2 Hz, Me), 3.90 (m, 1H, CH), 4.40 (m, 1H, CH), 4.71 (m, 4H, 2 × CH_2_), 4.90 (m, 1H, CH), 6.90 (s, 1H, CH), 7.20 (s, 1H, CH), 7.33-7.55 (m, 14H, CH, Ph), 7.65 (m, 2H, 10.90 (br s, NH).^13^C NMR (125.76 MHz, CDCl_3_): δ = 15.10, 69.4, 71.90, 72.28, 76.55, 111.07, 106.19, 127.91-129.14 (13 × C), 131.71, 132.79, 136.69, 137.33, 137.59, 139.29, 148.77, 150.91, 159.17, 170.51. MS (CI isobutane): *m/z* (%) = 511.19 (17) (M+). – C_30_H_29_N_3_O_3_S (511): calcd. C 70.43, H 5.71, N 8.21 found C 70.51, H 5.77, N 8.39.

### (2S,3S,4S)-3,4-bis(benzyloxy)-4-(1-(perfluorophenyl)-1H-pyrazol-4-yl)butan-2-ol (11)

Yield: 47 %, yellowish syrup, IR (NaCl): *v* = 1577 (C = C), 3255 (OH) ^1^HNMR (500.174 MHz, CDCl_3_): δ = 1.13 (d, 3H, *j* = 6.2 Hz, Me), 3.3 (m, 2H, CH_2_), 3.51 (dd, 1H, CH), 3.69 (m, 1H, CH), 4.19 (d, 1H, *j* = 11.8 Hz, CH), 4.33 (m, 2H, CH_2_), 4.37 (d, 1H, *j* = 11.8 Hz, CH), 4.39 (d, 1H, *j* = 12.2 Hz, CH), 4.89 (m, 2H, CH_2_), 7.13-7.29 (m, 10H, Ph), 7.61 (d, 1H, CH), 8.10 (d, 1H, *j* = 2.5 Hz, CH). ^13^C NMR (125.76 MHz, CDCl_3_): δ = 19.11, 67.10, 71.40, 72.61, 82.81, 90.58, 115.37, 125.70, 125.30-126.80 (10 × C), 136.91, 137.10, 137.88, 138.20, 138.71, 139.11, 140.20, 140.71. MS (CI isobutane): *m/z* (%) = 518.16 (11) (M+). – C_24_H_23_F_5_N_2_O_3_ (518): calcd. C 62.55, H 4.47, N 5.40 found C 62.49, H 4.40, N 5.48.

### (2S,3S,4S)-3,4-bis(benzyloxy)-4-(1-(pyridin-2-yl)-1H-pyrazol-4-yl)butan-2-ol (12)

Yield: 47 %, light yellow syrup, IR (NaCl): *v* = 1617 (C = N), 3334 (OH): ^1^HNMR (500.174 MHz, CDCl_3_): δ = d 1.09 (d, 3H, *j* = 6.5 Hz, Me), 2.39 (br, 1H, OH); 3.33 (dd, 2H, *j* = 5.5 Hz, CH_2_); 3.55 (m, 1H, CH); 4.27 (d, 1H, *j* = 11.8 Hz, CH), 4.45 (d, 1H, *j* = 11.2 Hz, CH), 4.50 (d, 2H, *j* = 11.1 Hz, CH); 7.08–7.30 (m, 13H, Ph); 7.43 (d, 1H, *j* = 1.5, CH), 7.84 (dd, 1H, CH); 8.14 (d, 1H, *j* = 2.4 Hz, CH). ^13^C NMR (125.76 MHz, CDCl_3_): δ = 20.1, 70.6, 71.1, 73.3, 79.7, 82.9, 93.1, 120.3, 122.6, 126.1–135.7 (13 × C), 136.2, 137.7, 143.6, 148.3, 150.8. MS (CI isobutane): *m/z* (%) = 535 (39) (M+). –C_26_H_27_N_3_O_3_ (429.21): calcd. C 72.71, H 6.34, N 9.78, found C 72.80, H 6.30, N 9.87.

### (E)-2-(((2S,3S,4S)-3,4-bis(benzyloxy)-2-methyl-3,4-dihydro-2H-pyran-5-yl)methylene)-hydrazinecarbothioamide (13)

Yield: 62 %, yellowish syrup, IR (NaCl): *v* = 1618 (C = N), 3208 (NH): ^1^HNMR (500.174 MHz, CDCl_3_): δ = 1.11 (d, 3H, *j* = 7.0 Hz, Me), 2.33 (br, 1H, OH); 3.32 (dd, *j* = 5.7 Hz, 2H, CH_2_); 3.55 (m, 1H, CH); 431 (m, 1H, CH), 4.41 (d, 1H, *j* = 11.2 Hz, CH), 4.52 (d, 2H, *j* = 11.1 Hz, CH); 7.11–7.26 (m, 11H, Ph); 7.84 (s, 1H, CH); 8.87 (s, 1H, NH); 10.82 (br s, 2H, NH_2_). ^13^C NMR (125.76 MHz, CDCl_3_): δ = 20.4, 70.2, 71.1, 72.9, 79.5, 82.7, 110.8, 125.4–135.2 (10 × C), 135.7, 137.4, 143.6, 147.7, 181.2. MS (CI isobutane): *m/z* (%) = 412 (24) (M+). –C_22_H_25_N_3_O_3_S (411.16): calcd. C 64.21, H 6.12, N 10.21, found C 64.55, H 6.19, N 10.32.

### (2S,3S,4S)-3,4-bis(benzyloxy)-4-(2-methylpyrazolo[1,5-a]pyrimidin-6-yl)butan-2-ol (14)

Yield: 47 %, yellowish syrup, IR (NaCl): *v* = 1628 (C = N), 3371 (OH): ^1^HNMR (500.174 MHz, CDCl_3_): δ = d 1.09 (d, 3H, *j* = 6.2 Hz, Me), 2.23 (s, 3H, Me); 2.41 (br, 1H, OH); 3.31 (m, 2H, CH_2_); 3.52 (m, 1H, CH); 4.25 (d, 1H, *j* = 11.2, CH), 4.41 (d, 2H, *j* = 11.2, CH), 4.53 (d, 1H, *j* = 11.0, CH); 7.11–7.27 (m, 10, Ph); 8.29 (s, 1H, CH); 8.41 (d, 2H, *j* = 2.1 Hz, 2 × CH). ^13^C NMR (125.76 MHz, CDCl_3_): δ = 14.1, 19.8, 70.3, 70.9, 73.1, 79.5, 83.2, 93.7, 125.5–132.1 (11 × C), 135.8, 137.2, 143.1, 147.9, 150.5, 155.5. MS (CI isobutane): *m/z* (%) = 417 (22) (M+). – C_25_H_27_N_3_O_3_ (417.21): calcd. C 71.92, H 6.52, N 10.06, found C 71.78, H 6.40, N 10.21.

## Author contributions

AB: Synthesized and purified the compounds; AA and AA-O: Provided the chemicals for all biological assays and helped in relevant literature; SA: Performed the mass spectrometric analysis and refined the manuscript; MM: drafted the manuscript, and refined for publication.

### Conflict of interest statement

The authors declare that the research was conducted in the absence of any commercial or financial relationships that could be construed as a potential conflict of interest.
