# Supplementary material for: Synthesis, Antiviral, and Antimicrobial Evaluation of Benzyl Protected Diversified C-nucleosides
Source: Front Chem. 2018 Jul 19;6:294. doi: 10.3389/fchem.2018.00294 (PMC6060234; doi:10.3389/fchem.2018.00294)
Supplement: Supplementary file 2 [file Data_Sheet_1.PDF]

N CDCl3 D:\\ aba

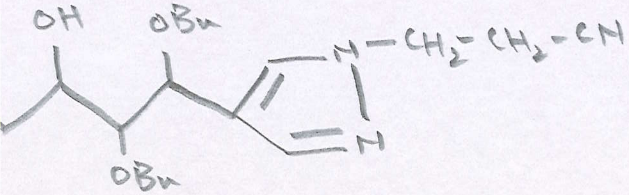

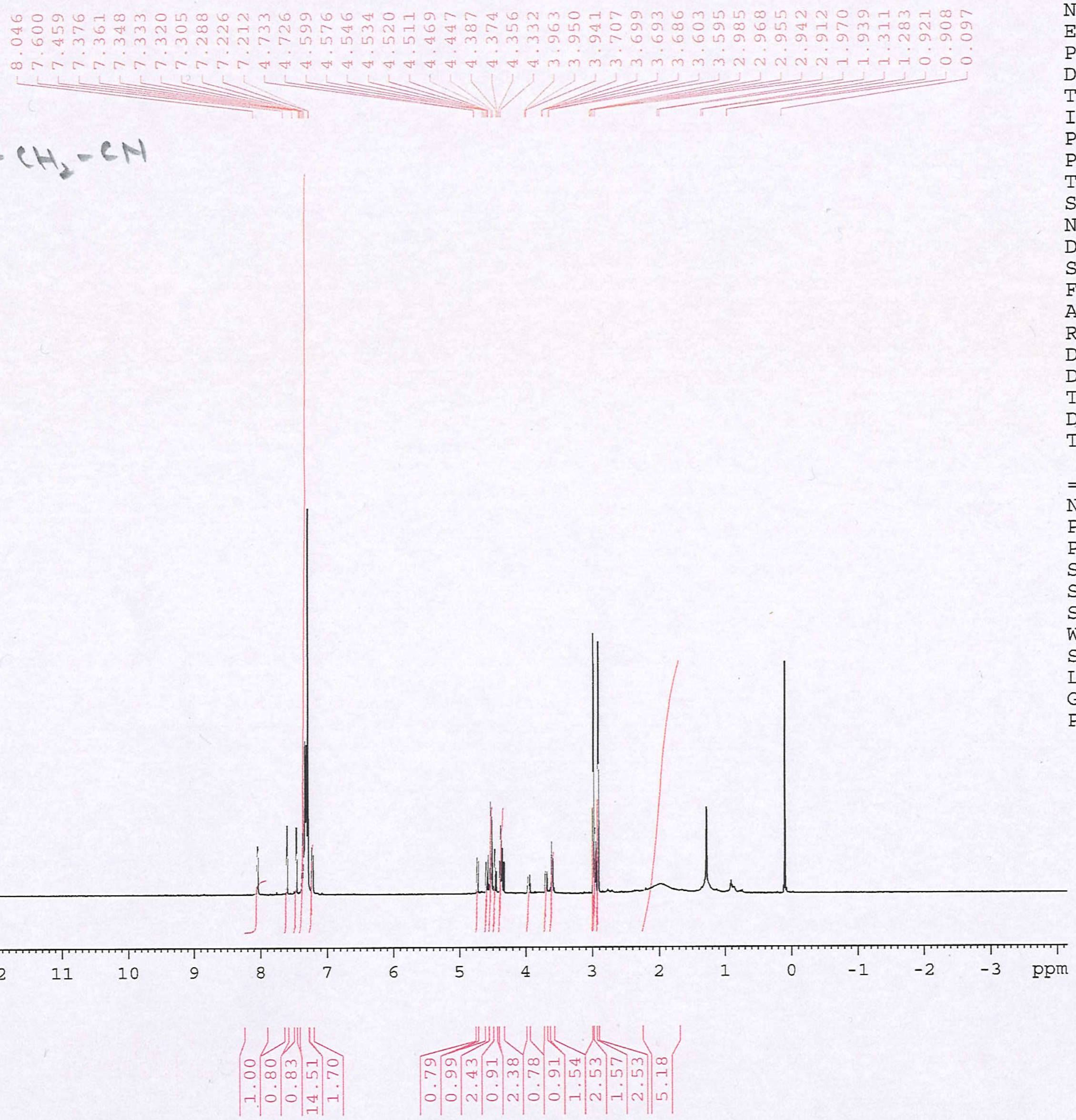

BRUKER

NAME	drbari-Med-9
EXPNO	10
PROCNO	1
Date_	20150330
Time	14.33
INSTRUM	spect
PROBHD	5 mm PABBO BB-
PULPROG	zg30
TD	65536
SOLVENT	CDCl3
NS	16
DS	2
SWH	10330.578 Hz
FIDRES	0.157632 Hz
AQ	3.1720407 sec
RG	362
DW	48.400 usec
DE	6.50 usec
TE	298.3 K
D1	1.00000000 sec
TD0	1

======== CHANNEL f1 ========

NUC1	1H
P1	14.70 usec
PL1	-1.10 dB
SFO1	500.1330885 MHz
SI	32768
SF	500.1300000 MHz
WDW	EM
SSB	0
LB	0.30 Hz
GB	0
PC	1.00

N CDCl3 D:\\ aba

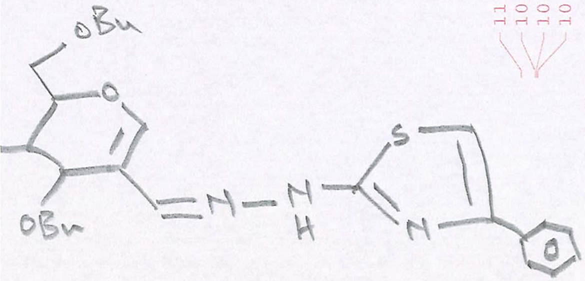

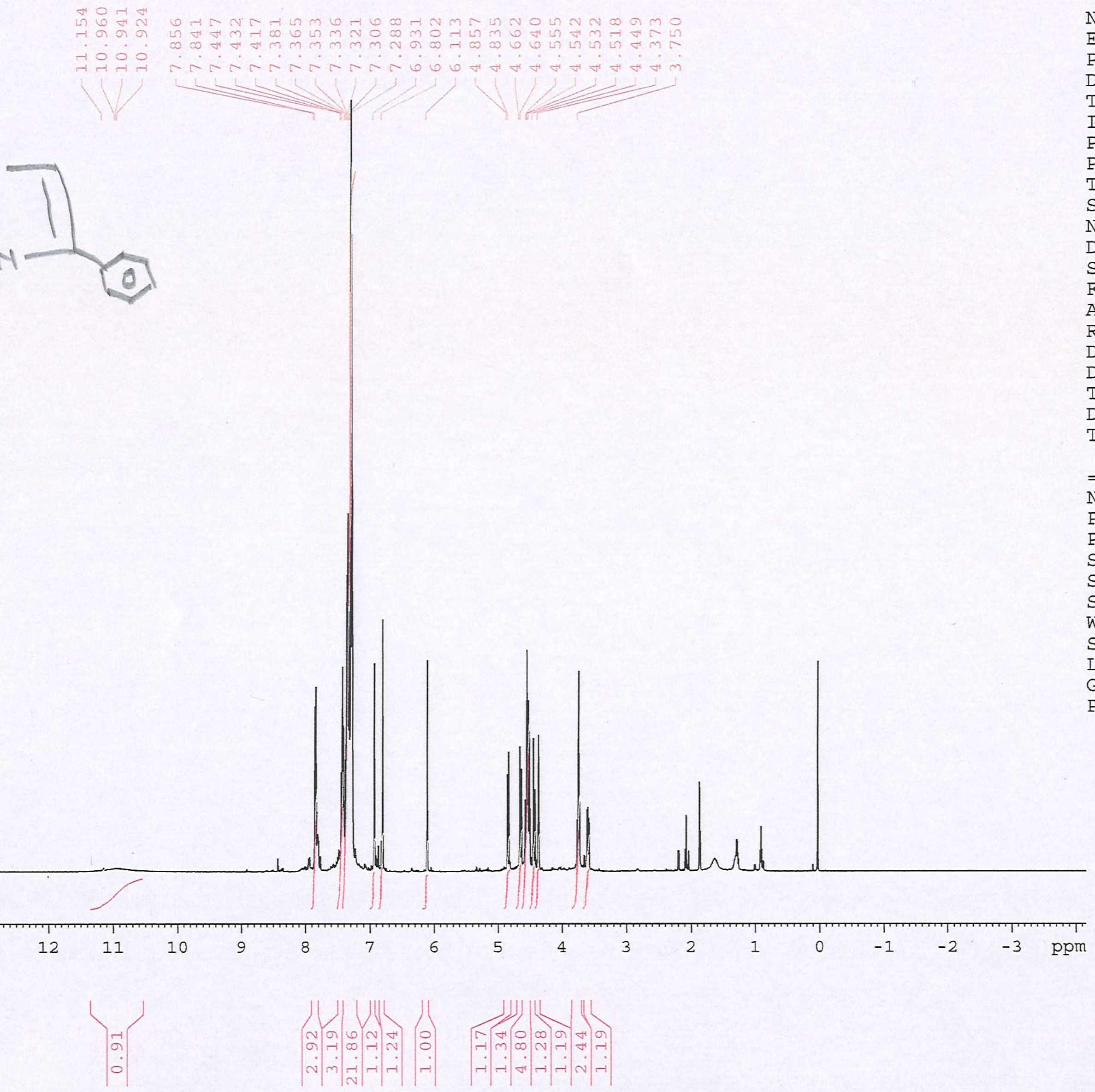

BRUKER

NAME	drbari-Med15-21
EXPNO	10
PROCNO	1
Date_	20150222
Time	15.48
INSTRUM	spect
PROBHD	5 mm PABBO BB-
PULPROG	zg30
TD	65536
SOLVENT	CDCl3
NS	16
DS	2
SWH	10330.578 Hz
FIDRES	0.157632 Hz
AQ	3.1720407 sec
RG	228.1
DW	48.400 usec
DE	6.50 usec
TE	297.0 K
D1	1.00000000 sec
TD0	1

======== CHANNEL f1 ========

NUC1	1H
P1	14.70 usec
PL1	-1.10 dB
SFO1	500.1330885 MHz
SI	32768
SF	500.1300000 MHz
WDW	EM
SSB	0
LB	0.30 Hz
GB	0
PC	1.00

ɪN CDCl3 D:\\ aba:

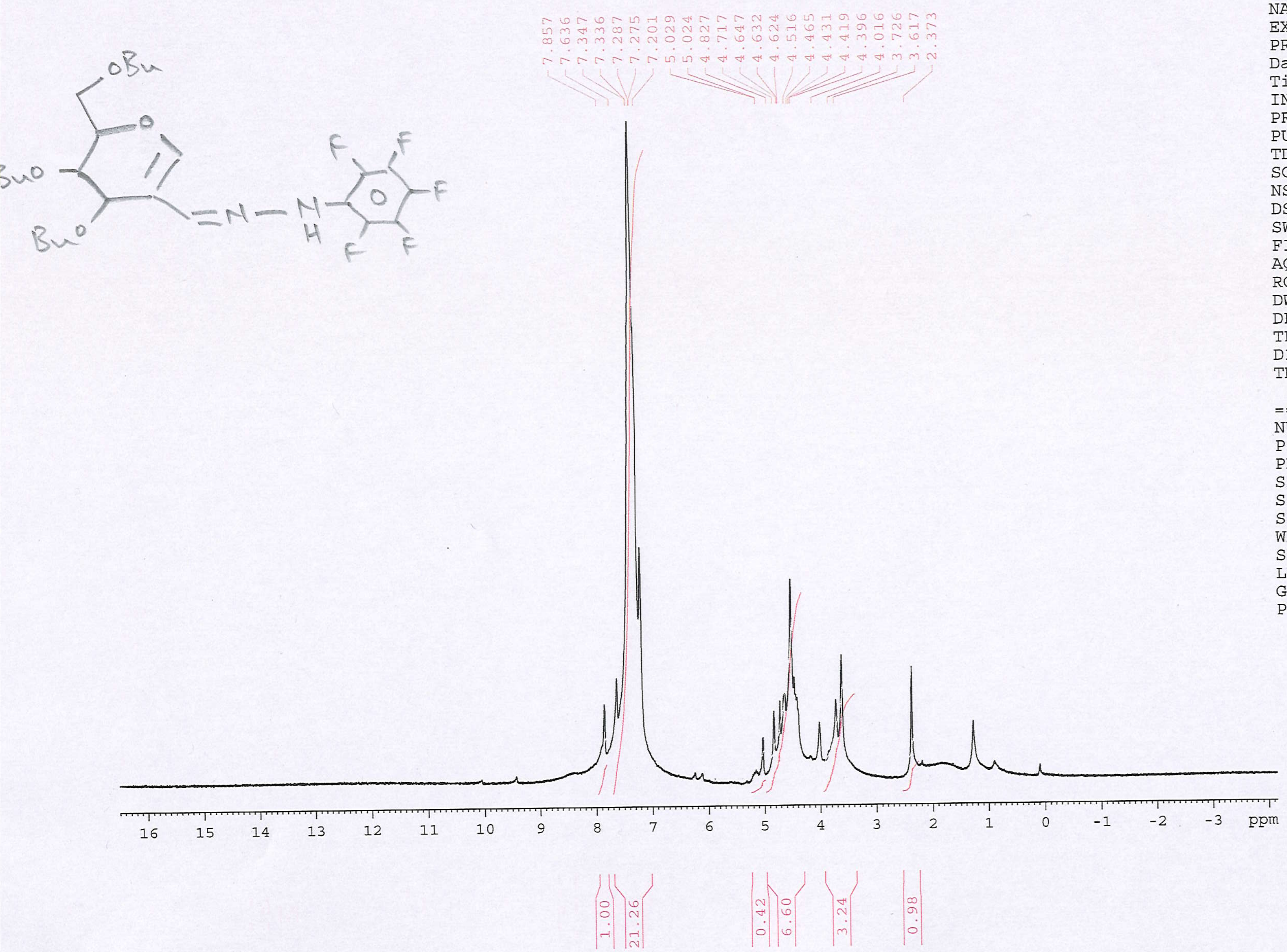

BRUKER

NAME	drbari-Med-1016-A
EXPNO	10
PROCNO	1
Date_	20160118
Time	11.57
INSTRUM	spect
PROBHD	5 mm PABBO BB-
PULPROG	zg30
TD	65536
SOLVENT	CDCl3
NS	16
DS	2
SWH	10330.578 Hz
FIDRES	0.157632 Hz
AQ	3.1720407 sec
RG	256
DW	48.400 usec
DE	6.50 usec
TE	296.4 K
D1	1.00000000 sec
TD0	1

======== CHANNEL f1 ========

NUC1	1H
P1	14.70 usec
PL1	-1.10 dB
SFO1	500.1330885 MHz
SI	32768
SF	500.1300000 MHz
WDW	EM
SSB	0
LB	0.30 Hz
GB	0
PC	1.00

'N CDCl3 D:\\ aba

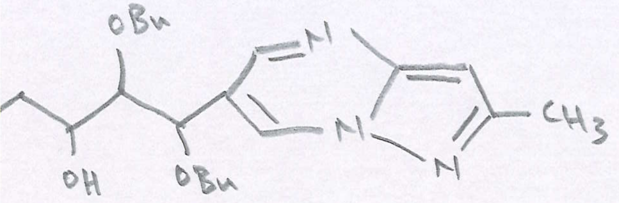

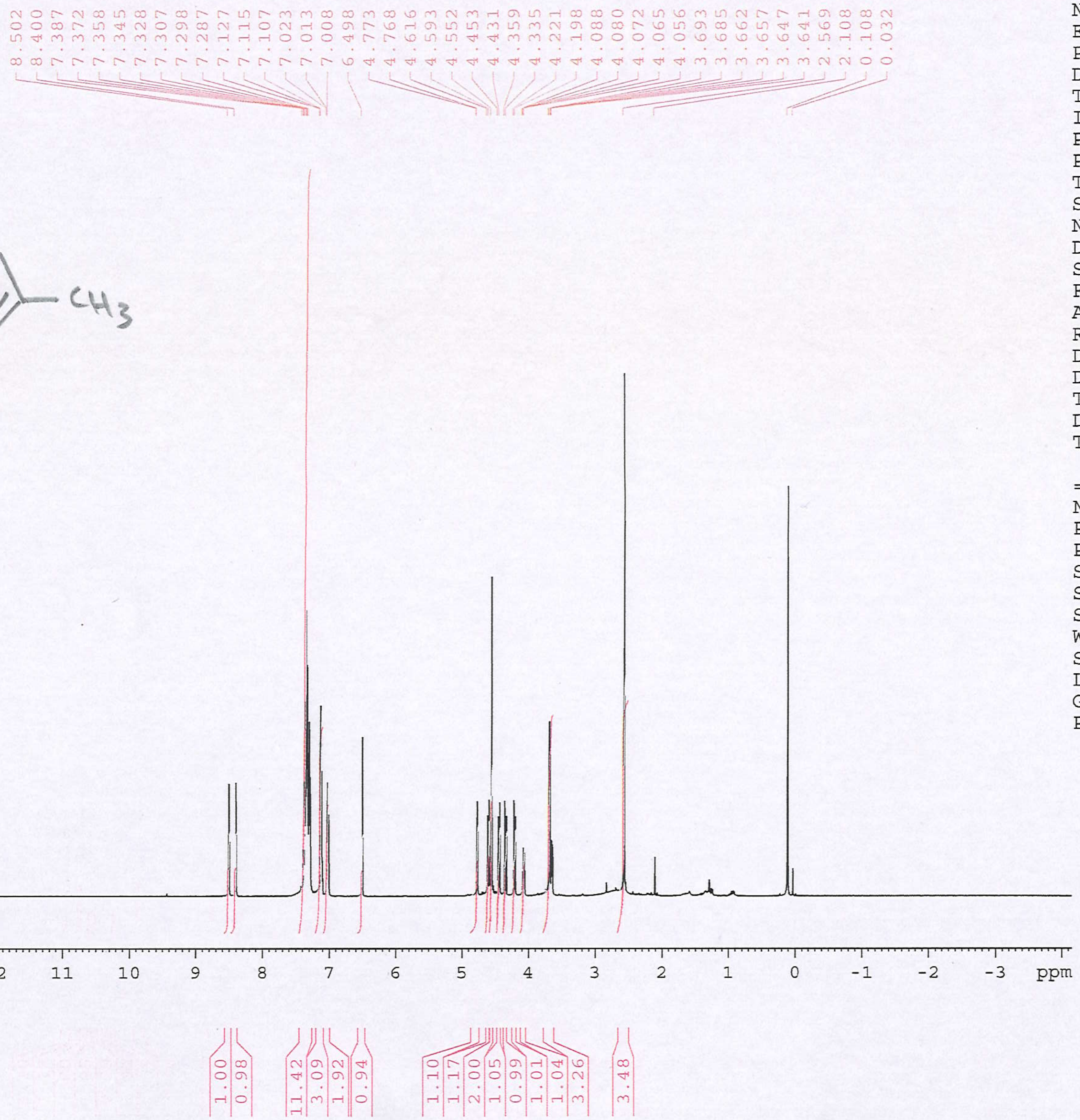

BRUKER

NAME	drbari-Med-20
EXPNO	10
PROCNO	1
Date_	20150513
Time_	12.00
INSTRUM	spect
PROBHD	5 mm PABBO BB-
PULPROG	zg30
TD	65536
SOLVENT	CDCl3
NS	16
DS	2
SWH	10330.578 Hz
FIDRES	0.157632 Hz
AQ	3.1720407 sec
RG	71.8
DW	48.400 usec
DE	6.50 usec
TE	297.8 K
D1	1.00000000 sec
TD0	1

======== CHANNEL f1 ========

NUC1	1H
P1	14.70 usec
PL1	-1.10 dB
SFO1	500.1330885 MHz
SI	32768
SF	500.1300000 MHz
WDW	EM
SSB	0
LB	0.30 Hz
GB	0
PC	1.00
