# Supplementary material for: Synthesis, Antiviral, and Antimicrobial Evaluation of Benzyl Protected Diversified C-nucleosides
Source: Front Chem. 2018 Jul 19;6:294. doi: 10.3389/fchem.2018.00294 (PMC6060234; doi:10.3389/fchem.2018.00294)
Supplement: Supplementary file 3 [file Data_Sheet_2.PDF]

Community for Open
Antimicrobial Drug Discovery

Primary Antimicrobial Screening

Bacterial and Fungal

Procedure and Materials

Content

1.0 Summary 3
1.1 Study 3
1.2 Assay Parameters 3
1.3 Outcomes 3
1.4 Comments 4
1.5 Publishing CO-ADD data 4
2.0 Methods 5
2.1 Sample preparation 5
2.2 Antimicrobial Assay 5
2.2.1 Procedure 5
2.2.2 Analysis 5
2.3 Antifungal Assay 5
2.3.1 Procedure 5
2.3.2 Analysis 6
2.4 Antibiotic standards preparation and Quality control 6
3.0 Materials 7
3.1 Assay materials 7
3.2 Standards 7
3.3 Microbial Strains 7
4.0 Controls 8

1.0 Summary

1.1 Study

Primary antimicrobial screening study by whole cell growth inhibition assays, using the provided samples at a single concentration, in duplicate (n=2). The inhibition of growth is measured against 5 bacteria: *Escherichia coli*, *Klebsiella pneumoniae, Acinetobacter baumannii, Pseudomonas aeruginosa* and *Staphylococcus aureus*, and 2 fungi: *Candida albicans* and *Cryptococcus neoformans*.

1.2 Assay Parameters

Test concentration	32 µg/mL or<br>20 µM<br>≤1% DMSO
QC	Duplicate (n=2)<br>Control MIC: Pass
Plates	Non-Binding Surface, 384 well plate
Media ***Bacteria***<br>***Fungi***	Cation-adjusted Mueller Hinton broth<br>Yeast Nitrogen Base
Read Out ***Bacteria***<br>***C. albicans***<br>***C. neoformans***	OD_{600}<br>OD_{530}<br>Resazurin $OD_{600-570}$

1.3 Outcomes

Primary Screening outcomes are detailed in individual Project reports, personalised for each Project Submission for each CO-ADD user.

Please see your data sheet with file extension **P0XXX_PS_data.xlsx,** for example CO-ADD Project **P0100, P0100_PS_data.xlsx**

1.4 Comments

To confirm the inhibitory activity, the hit compound/s will be re-tested against the strains in a dose response assay to determine the minimum inhibitory concentration (MIC) of the compounds. Furthermore, to further evaluate the antimicrobial potential of the compounds they will be assayed against a mammalian cell line to determine general cell toxicity.

In order to continue with Hit Confirmation assays, CO-ADD requests (as per the standard T&C's) that chemical structures of the compound/s (both active and inactive) be supplied after receipt of the primary screening report. All structural information will be kept confidential and only used internally by CO-ADD for the purpose of evaluating novelty of the chemistry to choose compounds for further validation. No publication will result without your written consent.

If you have not already provided structures to CO-ADD for your full compound set, please do so within a reasonable timeframe after receiving this report, so as not to delay Hit Confirmation.

1.5 Publishing CO-ADD data

If you wish to publish data provided by CO-ADD we kindly ask that you acknowledge CO-ADD appropriately with the following text:

"Antimicrobial screening was performed by CO-ADD (The Community for Antimicrobial Drug Discovery), funded by the Wellcome Trust (UK) and The University of Queensland (Australia)."

Please advise us at your earliest convenience that you have used provided data for publication purposes. This information is extremely helpful in keeping track of the outputs from the CO-ADD initiative and supports the program in renewed funding possibilities to continue CO-ADD as a free screening service available to the academic community.

2.0 Methods

2.1 Sample preparation

Samples were provided by the collaborator and stored frozen at -20 °C. Samples were prepared in DMSO and water to a final testing concentration of 32 µg/mL or 20 µM (unless otherwise indicated in the data sheet), in 384-well, non-binding surface plate (NBS) for each bacterial/fungal strain, and in duplicate (n=2), and keeping the final DMSO concentration to a maximum of 1% DMSO. All the sample-preparation where done using liquid handling robots.

Compounds that showed solubility issues during stock solution preparation are detailed in the data sheet.

2.2 Antimicrobial Assay

2.2.1 Procedure

All bacteria were cultured in Cation-adjusted Mueller Hinton broth (**CAMHB**) at 37 °C overnight. A sample of each culture was then diluted 40-fold in fresh broth and incubated at 37 °C for 1.5-3 h. The resultant mid-log phase cultures were diluted (CFU/mL measured by OD_{600}), then added to each well of the compound containing plates, giving a cell density of 5×10^5 CFU/mL and a total volume of 50 µL. All the plates were covered and incubated at 37 °C for 18 h without shaking.

2.2.2 Analysis

Inhibition of bacterial growth was determined measuring absorbance at 600 nm (OD_{600}), using a Tecan M1000 Pro monochromator plate reader. The percentage of growth inhibition was calculated for each well, using the negative control (media only) and positive control (bacteria without inhibitors) on the same plate as references. The significance of the inhibition values was determined by modified Z-scores, calculated using the median and MAD of the samples (no controls) on the same plate. Samples with inhibition value above 80% and Z-Score above 2.5 for either replicate (n=2 on different plates) were classed as actives. Samples with inhibition values between 50 - 80% and Z-Score above 2.5 for either replicate (n=2 on different plates) were classed as partial actives. Samples with inhibition values between 50 - 80% and Z-Score above 2.5 for either replicate (n=2 on different plates) were classed as partial actives.

2.3 Antifungal Assay

2.3.1 Procedure

Fungi strains were cultured for 3 days on Yeast Extract-Peptone Dextrose (**YPD**) agar at 30 °C. A yeast suspension of 1×10^6 to 5×10^6 CFU/mL (as determined by OD_{530}) was prepared from five colonies. The suspension was subsequently diluted and added to each well of the compound-containing plates giving a final cell density of fungi suspension of 2.5×10^3 CFU/mL and a total volume of 50 µL. All plates were covered and incubated at 35 °C for 24 h without shaking.

2.3.2 Analysis

Growth inhibition of *C. albicans* was determined measuring absorbance at 530 nm (OD_{530}), while the growth inhibition of *C. neoformans* was determined measuring the difference in absorbance between 600 and 570 nm ($OD_{600-570}$), after the addition of resazurin (0.001% final concentration) and incubation at 35 °C for additional 2 h. The absorbance was measured using a Biotek Synergy HTX plate reader. The percentage of growth inhibition was calculated for each well, using the negative control (media only) and positive control (fungi without inhibitors) on the same plate. The significance of the inhibition values was determined by modified Z-scores, calculated using the median and MAD of the samples (no controls) on the same plate. Samples with inhibition value above 80% and Z-Score above 2.5 for either replicate (n=2 on different plates) were classed as actives. Samples with inhibition values between 50 - 80% and Z-Score above 2.5 for either replicate (n=2 on different plates) were classed as partial actives.

2.4 Antibiotic standards preparation and Quality control

Colistin and Vancomycin were used as positive bacterial inhibitor standards for Gram-negative and Gram-positive bacteria, respectively. Fluconazole was used as a positive fungal inhibitor standard for *C. albicans and C. neoformans.*

The antibiotics were provided in 4 concentrations, with 2 above and 2 below its MIC value, and plated into the first 8 wells of column 23 of the 384-well NBS plates.

The quality control (QC) of the assays was determined by the antimicrobial controls and the Z'-factor (using positive and negative controls). Each plate was deemed to fulfil the quality criteria (pass QC), if the Z'-factor was above 0.4, and the antimicrobial standards showed full range of activity, with full growth inhibition at their highest concentration, and no growth inhibition at their lowest concentration.

3.0 Materials

3.1 Assay materials

Material	Code	Brand	Cat No.
Compound preparation plate	PP	Corning	3364
Assay Plates	NBS 384w	Corning	3640
Growth media - bacteria	CAMHB	Bacto Laboratories	212322
Culture agar - fungi	YPD	Becton Dickinson	242720
Growth media - fungi	YNB	Becton Dickinson	233520
Resazurin		Sigma-Aldrich	R7017

3.2 Standards

Sample Name	Sample ID	Full MW	Stock Conc (mg/mL)	Solvent	Source
Colistin - Sulfate	MCC_000094:02	1400.63	10.0	DMSO	Sigma; C4461
Vancomycin - HCL	MCC_000095:02	1485.71	10.0	DMSO	Sigma; 861987
Fluconazole	MCC_008383:01	306.27	2.56	DMSO	Sigma; F8929

3.3 Microbial Strains

ID	Batch	Organism	Strain	Description
GN_001	02	*Escherichia coli*	ATCC 25922	FDA control strain
GN_003	02	*Klebsiella pneumoniae*	ATCC 700603	MDR
GN_034	02	*Acinetobacter baumannii*	ATCC 19606	Type strain
GN_042	02	*Pseudomonas aeruginosa*	ATCC 27853	Quality control strain
GP_020	02	*Staphylococcus aureus*	ATCC 43300	MRSA
FG_001	01	*Candida albicans*	ATCC 90028	CLSI reference
FG_002	01	*Cryptococcus neoformans*	ATCC 208821	H99 - Type strain

4.0 Controls

All antibiotic controls displayed inhibitory values within the expected range.

Strain ID	***Species***	***Antibiotic***	***Pass/Fail***
GN_001:02	*E. coli*	Colistin	Pass
GN_003:02	*K. pneumoniae* (MDR)	Colistin	Pass
GN_034:02	*A. baumannii*	Colistin	Pass
GN_042:02	*P. aeruginosa*	Colistin	Pass
GP_020:02	*S. aureus* (MRSA)	Vancomycin	Pass
FG_001:01	*C. albicans*	Fluconazole	Pass
FG_002:01	*C. neoformans* (H99)	Fluconazole	Pass
